# Retraction: Sasidharan *et al*. Evaluation of the Hepatoprotective Effects of Lantadene A, a Pentacyclic Triterpenoid of *Lantana* Plants against Acetaminophen-induced Liver Damage. *Molecules* 2012, *17*, 13937-13947

**DOI:** 10.3390/molecules18043839

**Published:** 2013-03-27

**Authors:** Sreenivasan Sasidharan

**Affiliations:** Institute for Research in Molecular Medicine (INFORMM), Universiti Sains Malaysia, Pulau Pinang 11800, Malaysia; E-Mail: srisasidharan@yahoo.com

A recent *Comment* by M. Sharma published in the journal *Molecules* [1] raises issues with our previously published paper [2]. After reviewing its content, and although we respectfully stand by our experimental description whereby we were able to prepare stock and working solutions of the substance being tested, the arguments presented do raise concerns about the true identity of the compound actually used and hence the results and conclusions of our paper.

Unfortunately, since becoming aware of this, we have been unable to characterize the original sample, now used up, and we have been unable to obtain a verified equivalent one from the local commercial supplier we used. In light of these facts, since we can no longer associate with any confidence our experimental results with the compound lantadene A, we would like to withdraw our paper until such a time that we can obtain a new sample, check its identity and redo the work. As to the comments regarding erroneous citation of references, we feel that since the paper is to be removed from the scientific record, any additional corrections are unnecessary at this time. As corresponding author I take full responsibility for the omission of not checking the compound used in our experiments and any errors in its contents, and would like to offer my apologies on behalf of my co-authors to the readership of *Molecules* for any inconveniences caused by this omission.
